# c-kit^+^VEGFR-2^+^ Mesenchymal Stem Cells Differentiate into Cardiovascular Cells and Repair Infarcted Myocardium after Transplantation

**DOI:** 10.1007/s12015-022-10430-z

**Published:** 2022-08-13

**Authors:** Pei Zhou, Shu-na Yu, Hai-feng Zhang, Yong-li Wang, Ping Tao, Yu-zhen Tan, Hai-jie Wang

**Affiliations:** Department of Anatomy, Histology and Embryology, Shanghai Medical School of Fudan University, 138 Yixueyuan Road, Shanghai, 200032 People’s Republic of China

**Keywords:** c-kit, VEGFR-2, Mesenchymal stem cells, Stem cell transplantation, Angiogenesis, Myocardial regeneration, Myocardial infarction

## Abstract

Resent study suggests that c-kit^+^ cells in bone marrow-derived MSCs may differentiate toward cardiamyocytes. However, the properties of c-kit^+^ MSCs remain unclear. This study isolated c-kit^+^VEGFR-2^+^ cells from rat bone marrow-derived MSCs, and assessed potential of c-kit^+^VEGFR-2^+^ MSCs to differentiate towards cardiovascular cells and their efficiency of repairing the infarcted myocardium after transplantation. Gene expression profile of the cells was analyzed with RNA-sequencing. Potential of differentiation of the cells was determined after induction. Rat models of myocardial infarction were established by ligation of the left anterior descending coronary artery. The cells were treated with hypoxia and serum deprivation for four hours before transplantation. Improvement of cardiac function and repair of the infarcted myocardium were assessed at four weeks after transplantation. Gene expression profile revealed that c-kit^+^VEGFR-2^+^ MSCs expressed most smooth muscle-specific and myocardium-specific genes, while expression of endothelium-specific genes was upregulated significantly. After induction with VEGF or TGF-β for two weeks, the cells expressed CD31 and α-SMA respectively. At three weeks, BMP-2-induced cells expressed cTnT. After transplantation of the cells, cardiac function was improved, scar size of the infarcted myocardium was decreased, and angiogenesis and myocardial regeneration were enhanced significantly. Moreover, paracrine in the myocardium was increased after transplantation. These results suggest that c-kit^+^VEGFR-2^+^ MSCs have a potential of differentiation towards cardiovascular cells. Transplantation of c-kit^+^VEGFR-2^+^ MSCs is effective for repair of the infarcted myocardium. c-kit^+^VEGFR-2^+^ MSCs may be a reliable source for cell therapy of ischaemic diseases.

**Graphical abstract:**

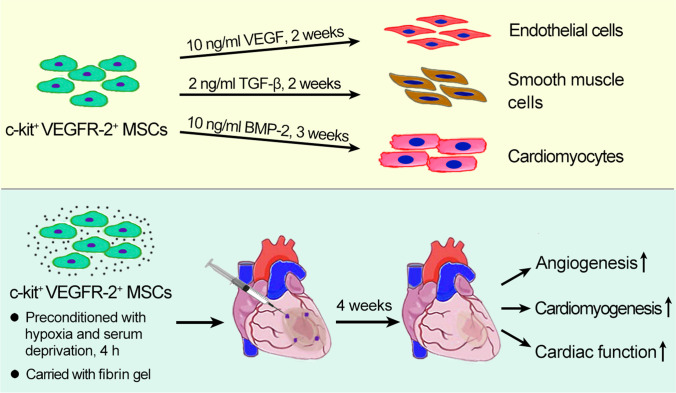

**Supplementary Information:**

The online version contains supplementary material available at 10.1007/s12015-022-10430-z.

## Introduction

Myocardial infarction (MI) is a leading cause of death of the cardiovascular diseases worldwide. The overall prevalence for MI is 2.8% in US adults. MI prevalence is 4.0% for men and 1.8% for women [1]. According to investigation of the European Society of Cardiology, one in six men and one in seven women in Europe will die from MI. However, despite improvements in pharmacological and interventional treatments, one in three men and one in four women die within a year of their first MI [2].

After the occlusion of a coronary artery, the resulting ischaemia can be severe leading to the death of both cardiomyocytes and non-myocytes locally, followed by local inflammation, degradation of extracellular matrix, scar formation and remodelling of the ventricular wall. The heart with MI may lose one billion cardiomyocytes, approximately 25% of its mass [3]. The current approach to MI treatment involves early revascularisation with percutaneous coronary intervention or coronary artery bypass grafting, followed by the medical management of atherosclerotic risk factors, late ventricular remodelling and cardiac arrhythmias. While the mammalian heart appears capable of endogenous regeneration at the early neonatal stage, this capacity is lost shortly after birth [4]. In human heart, renewal of cardiomyocytes is up to 1% per year at the age of 25 to 0.45% at the age of 75 [5]. Over the past decade, stem cell transplantation has emerged as a promising therapy for myocardial regeneration [6]. In several candidate stem cells, cardiac stem cells (CSCs) and mesenchymal stem cells (MSCs) have been considered as safe and reliable cell populations for cardiac transplantation [7].

c-kit^+^ CSCs are characterized by a potential to differentiate toward cardiovascular cells though cardiomyogenic potential is controversial [8]. c-kit (as known as CD117) is a tyrosine kinase receptor that activates a downstream signaling cascade on binding to stem cell factor (SCF). c-kit regulated cardiomyocyte terminal differentiation and promoted CSC differentiation [9]. c-kit^+^Nkx2.5^+^ cells isolated from mouse embryonic heart possessed the capacity for differentiation into cardiomyocytes and smooth muscle cells [10]. Lin^−^c-kit^+^ cells isolated from rat heart [11] and human myocardial sample of cardiac surgery [12] are self-renewing, clonogenic and multipotent, giving rise to cardiomyocytes, endothelial cells, and smooth muscle cells. The number of c-kit^+^Nkx2.5^+^GATA4^+^ cells increased significantly after cardiac injury [13]. Neonatal c-kit^+^ cells had a cardiomyogenic potential, while this ability was lost in adult c-kit^+^ cells [14]. Endogenous c-kit cells minimally contribute to cardiomyogenesis during neonatal and adult heart regeneration [15, 16]. Interestingly, transplantation of Lin^−^c-kit^+^ CSCs [11] or CD45^−^c-kit^+^ CSCs [17] was resulted in robust cardiomyogenesis and angiogenesis in the infarcted myocardium. It is worth noting that c-kit, in itself, does not define a specific marker for CSCs, and also expressed on other cells such as mast cells [18] and hematopoietic cells [19]. Lin^−^CD45^−^c-kit^+^ cells only represented ≤ 10% of the adult rat c-kit^+^ cardiac cells [17]. If resident CSCs are identified with c-kit expression alone, their potential to differentiate towards cardiovascular cells and effects in cardiomyocytic regeneration is probably misestimated.

MSCs are relatively easy to be isolated from bone marrow or adipose tissue and can be expanded significantly ex vivo, exhibiting the properties of low immunogenicity and immunosuppression [20]. Regardless of the debated potential to generate cardiomyocytes in vivo, most studies suggest that MSCs may differentiate towards cardiomyocytes, vascular smooth muscle and endothelial cells. Preclinical and clinical trials have proved that MSCs can improve cardiac function and structure [21, 22]. Recent studies have suggested that there is a population of c-kit^+^ cells having a cardiac regenerative potential in bone marrow cells. Intramyocardial injection of Lin^−^c-kit^+^ bone marrow cells promoted myocardial regeneration, ameliorating the outcome of MI [23]. After intracoronary injection of autologous c-kit^+^ mononuclear cells isolated from bone marrow, cardiac function was improved, and pathological remodeling was attenuated [24]. c-kit^+^ cells isolated from bone marrow-derived MSCs had a potential to differentiate towards cardiomyocytes. Preinduction with bone morphogenetic protein-2 (BMP-2) enhanced cardiomyogenic differentiation of c-kit^+^ MSCs and repair of infarcted myocardium [25]. However, properties of c-kit^+^ MSCs and their effect on myocardium repair need further investigation.

This investigation was designed to sort c-kit-positive and VEGFR-2 (vascular endothelial growth factor receptor-2)-positive cells from rat bone marrow-derived MSCs, and assess biological characteristics of c-kit^+^VEGFR-2^+^ MSCs and evaluate their effects on repairing the infarcted myocardium after transplantation. Here, we demonstrate that c-kit^+^VEGFR-2^+^ MSCs have a potential of differentiation towards cardiovascular cells. c-kit^+^VEGFR-2^+^ MSCs can effectively repair the infarcted myocardium through their differentiation and paracrine after transplantation.

## Methods

### Isolation of c-kit^+^VEGFR-2^+^ MSCs

Isolation of MSCs from bone marrow of Sprague–Dawley (SD) rats (2 − 4 weeks) was performed as described previously [26] with minor modification. Briefly, bone marrow cells in the femurs and tibias were flushed out with PBS supplemented with 5 mM ethylene diamine tetraacetic acid (EDTA). The mononuclear cells in the bone marrow cells were isolated by Percoll (GE Healthcare, Leics, UK) gradient centrifugation, and then incubated with Dulbecco's modified Eagle's medium (DMEM; Hyclone, Logan, UT, USA) supplemented with 15% fetal bovine serum (FBS; Carlsbad, CA, USA) in 35-mm plastic dishes for 24 h. The non-adherent cells were discarded by vigorous washing, and the adherent cells continued to be incubated with complete medium as MSCs. The isolated MSCs were positive for mesenchymal lineage markers and negative for hematopoietic markers. MSCs at the second passage were digested using 0.25% trypsin–EDTA (Thermo Fisher Scientific, Waltham, MA, USA) for 10 min at 37 °C and then resuspended in 1% bovine serum albumin (BSA, Amresco, Solon, OH, USA). After centrifugation, the cells were incubated with mouse anti-rat c-kit antibody (1:100) and polyclonal rabbit anti-rat VEGFR-2 antibody (1:200, Santa Cruz Biotechnology, Dallas, TX, USA) for 50 min at 4 °C. The cells were washed with 0.5% BSA and then incubated with Alexa Fluor 488-conjugated goat anti-rabbit IgG (1:200) and Alexa Fluor 647-conjugated goat anti-mouse IgG (1:400; Jackson ImmunoResearch Laboratories, West Grove, PA, USA) for 30 min at 4 °C. After washing twice with PBS, the cells were suspended with DMEM containing 2.5% FBS, and c-kit^+^VEGFR-2^+^ MSCs and c-kit^+^VEGFR-2^−^ MSCs were sorted respectively by a fluorescence-activated cell sorter (Beckman Coulter, Fullerton, CA, USA). The sorted cells were incubated with the complete medium, and the medium was changed every 3 days. c-kit^+^VEGFR-2^+^ MSCs and c-kit^+^VEGFR-2^−^ MSCs of the third to sixth passages were used in the following experiments.

### RNA-sequencing

For examining biologic characteristics of c-kit^+^VEGFR-2^+^ MSCs, total RNA of c-kit^+^VEGFR-2^+^ and c-kit^+^VEGFR-2^−^ MSCs was extracted using the TRIzol Reagent Kit (Invitrogen) respectively. The samples were processed following the manufacturer’s instructions (BGI Tech Solutions, Shenzhen, China). In brief, the target RNA was obtained after purification by mRNA enrichment using Oligo (dT) magnetic beads. Fragment target RNA was reversely transcribed into double-strand cDNA. After end repair and bubble adapter ligation, cDNA was amplified. PCR product was denatured by heat and cyclized to establish DNA library. Primary sequencing data (raw data) was acquired by Illumina HiSeq 2000 (Illumina, Santiago, CA, USA). After filtering raw data to clean data, reads were aligned to genome reference. Bowtie2 was used to map clean reads to Rattus norvegicus. Differentially expressed genes were screened using Possion distribution with the fragments assigned per kilobase of target per million mapped reads (FPKM) method. Moreover, the differentially expressed genes were identified with RT-PCR. The sequences of the primers were shown in Table S1. Gene ontology (GO) was used to recognize the main biological functions of the two subpopulations of c-kit^+^ cells. In addition, GO functional annotation and enrichment analysis was used to compare the molecular functions and biological processing of the cells in two subpopulations. The pathways enriched with differentially expressed genes were made with Database of Kyoto Encyclopedia of Genes and Genomes (KEGG) [27].

### Examination of Proliferation and Migration of the Cells

For proliferative assay, c-kit^+^VEGFR-2^+^ MSCs were divided into vehicle, VEGF (vascular endothelial growth factor), SCF and VEGF + SCF groups. In VEGF and SCF groups, 10 ng/ml VEGF (Peprotech, Rocky Hill, NJ, USA) and 50 ng/ml SCF (Peprotech) were added in the medium respectively. In VEGF + SCF group, 10 ng/ml VEGF and 50 ng/ml SCF were added in the medium simultaneously. After treatment with the growth factors for 24 h, the cells were incubated with rabbit anti-rat ki67 antibody (1:200; Abcam, Cambridge, UK), followed by Alexa Fluor 488-conjugated goat anti-rabbit IgG (1:200). Ki67-positive cells were counted with a fluorescence microscope.

Migration of the cells was assessed by transwell assay. The experimental grouping is the same as above. The diameter of the pores of the polyethylene terephthalate membrane in the transwell (Becton Dickinson, Franklin Lakes, NJ, USA) is 8 μm. The cells were seeded on the upper chamber, and the medium supplemented with the growth factors was added into the lower chamber. After incubation for 12 h, the cell insert was taken out and stained with 10% Giemsa. The cells on the upper chamber were wiped with tissue paper, and then the cells migrated into the lower chamber were counted. Six repeated experiments were performed.

### Tube Formation Assay

The incorporation capacity of the c-kit^+^VEGFR-2^+^ cells into tube-like structures formed by pulmonary microvascular endothelial cells was assessed using previous method [28]. The lungs of SD rats (two-weeks old) were removed and put into pre-iced HBSS. After removing the pleura, the tissue near the border of the lung was cut into 1 mm^3^ pieces. The tissue masses were incubated with DMEM in gelatin-coated wells for one week. The migrated endothelial cells were harvested, and then incubated in 24-well plate coated with Matrigel (3:2 in DMEM; BD Biosciences, San Jose, CA, USA). After capillary-like structures were formed, c-kit^+^VEGFR-2^+^ cells labelled with Dil (Beyotime Biotech, Haimen, Jiangsu, China) were added, and continued to be incubated for 4 h. c-kit^+^VEGFR-2^+^ cells incorporated into the capillary-like structures were counted with a fluorescence microscope. Five fields were selected randomly in each group. The experiment was repeated for four times. In VEGF group, c-kit^+^VEGFR-2^+^ cells were pretreated with 10 ng/ml VEGF for 2 h.

### Induction of Differentiation of c-kit^+^VEGFR-2^+^ MSCs towards Cardiovascular Cells

For assessing differentiation of c-kit^+^VEGFR-2^+^ MSCs towards endothelial cells, smooth muscle cells or cardiomyocytes, the cells were induced with 10 ng/ml VEGF, 2 ng/ml TGF-β (transforming growth factor-β; Peprotech) or 10 ng/ml BMP-2 (Peprotech) respectively. After induction for 2 weeks, expression of *CD31* and *vWF* (*von Willebrand factor*), *α-SMA* (*α-smooth muscle actin*) and *CNN1* (*calponin 1*), and *Nkx2.5* and *GATA-4* was analyzed by RT-PCR respectively. Total RNA was collected with the TRIzol Reagent Kit. After mRNA were reverted to cDNA using Primescript RT Reagent Kit with gDNA Eraser (Takara Biotechnology, Otsu, Japan), cDNA was amplified with PCR assay. The PCR products were separated on a 1.2% agarose gel electrophoresis and visualized under ultraviolet light. mRNAs of the femoral artery from SD rat were used as positive controls in VEGF and TGF-β groups, while mRNAs of the myocardium from SD rat were used as positive controls in BMP-2 group. The experiment was repeated for three times. The sequences of the primers were shown in Table S1. Moreover, expression of CD31 or α-SMA in VEGF and TGF-β groups was examined with immunostaining respectively. In cTnT immunostaining, the cells were induced with BMP-2 for 3 weeks. The cells were incubated with mouse anti-rat CD31 antibody, rabbit anti-rat α-SMA antibody (1:200; Abcam), or mouse anti-rat cTnT (cardiac troponin T) antibody (1:200; Santa Cruz Biotechnology) overnight, followed with Alexa Fluor 594-conjugated goat anti-mouse IgG (1:400) or Alexa Fluor 488-conjugated goat anti-rabbit IgG (1:200; Jackson ImmunoResearch) respectively. Expression of CD31, α-SMA and cTnT of the differentiated cells was viewed with a fluorescence microscope.

### Determination of Optimal Preconditioning Time

To mimic the hypoxic and ischemic microenvironment of the myocardium, c-kit^+^VEGFR-2^+^ MSCs were treated with hypoxia (1% O_2_) and serum deprivation (3% FBS) for 1 h, 2 h, 4 h, 6 h and 8 h. Then, the degrees of autophagy and apoptosis of the cells were assessed with Western blotting and flow cytometry respectively. After treatment, total protein in each group was extracted with RIPA buffer (Beyotime, Beijing, China) and separated in a 15% SDS–polyacrylamide gel. The samples were then transferred to PVDF membranes. After being blocked with 5% skim milk, the membranes were incubated with polyclone rabbit anti-rat LC3 (microtubule-associated protein 1 light chain 3) antibody (1:500; Novus, Littleton, CO, USA) and mouse anti-rat β-actin monoclonal antibody (1:4000; Proteintech, Rosemont, IL, USA) at 4 °C overnight. Then, they were incubated with HRP-linked anti-rabbit IgG and HRP-linked anti-mouse IgG (1:4000; Cell Signaling, Danvers, MA, USA) at room temperature for 1 h. After being washed with TBST, the protein bands were monitored using Substrate Chemiluminescence Kit (Thermo, Rockford, IL, USA). The ratio of LC3-II/β-actin was analyzed using ImageJ (National Institutes of Health, Bethesda, MD, USA). The experiment was repeated for four times. For examining apoptosis of the hypoxia-treated cells, the cells were labeled with FITC Annexin V Apoptosis Detection Kit (BD Biosciences). The percentage of the apoptotic cells was determined by flow cytometry. The experiment was repeated for thrice.

### Implantation of the Preconditioned Cells into Abdominal Pouches

To assess the survival of the cells preconditioned with hypoxia and serum deprivation in the ischaemic tissue, the cells were implanted into abdominal subcutaneous pouches of SD rats. The pouches were prepared as previous method [29]. To mimic the ischaemic tissue, the subcutaneous vessels at the pouch were ligated. After treatment with 5 μM DiI for 20 min, the cells were suspended with 20 ng/ml fibrinogen and 50 IU/ml thrombin, and seeded on the poriferous polyethylene terephthalate membrane (pore size, 8 μm) removed from the transwell. For formation of fibrin gel, the cell-loaded membranes were incubated for 20 min. Then, the membranes were implanted into the abdominal pouches. At 24 h after implantation, the membranes were harvested, and the survived cells (DiI-labelled cells) were counted with a fluorescence microscope. Five fields of each membrane were selected randomly. The experiment was repeated for six times.

### Enzyme-linked Immunosorbent Assay (ELISA)

For assessing paracrine of c-kit^+^VEGFR-2^+^ MSCs in the condition of ischemia and hypoxia, the cells were incubated with 1% O_2_ and 3% FBS for 12 h. Concentrations of VEGF, SCF and SDF-1α (stromal cell-derived factor-1α) in the supernatant were detected using ELISA kits (Boster, Wuhan, China) according to the manufacturer’s protocols. Absorbance was measured with a microplate reader (Tecan Infinite 200, Mannedorf, Switzerland) at 450 nm. Moreover, paracrine of VEGF, SCF and SDF-1α in plasma and myocardium at the peri-infarct region of the left ventricle (LV) was also examined using ELISA at 1 week after cell transplantation in rat MI models. In brief, LV tissues were cut into pieces and ground into tissue homogenate in 0.01 M PBS at a ratio of 1 g tissue to 9 ml PBS with homogenizer. After centrifugation, the supernatants were collected and stored at –20 °C. The paracrine factors secreted from the infarcted myocardium were detected with ELISA kits. The experiment was repeated for six times.

### Scanning and Transmission Electron Microscopies

To assess compatibility of fibrin with implanted cells, the fibrin gel was prepared by mixing 500 μl of fibrinogen (10 mg/ml) and 500 μl of thrombin (10 IU/ml; Sigma-Aldrich, St. Louis, MO, USA) in a 35-mm dish. The cells were seeded on the fibrin gel and incubated for 1 h. Then, the specimens were pre-fixed with 1.25% glutaraldehyde and post-fixed with 1% buffered osmium tetroxide. After dehydration and substitution, the specimens were coated with gold-palladium and then viewed with a scanning electron microscope (Hitachi SU8010, Tokyo, Japan). In transmission electron microscopy, the specimens were fixed with 2.5% glutaraldehyde, post-fixed with 1% buffered osmium tetroxide and dehydrated with graded ethanol and acetone. The ultrathin sections were stained with uranyl acetate and lead citrate. The cells within the fibrin gel were viewed using a transmission electron microscope (CM120; Philips, Eindhoven, Holland). Moreover, compatibility of fibrin with the transplanted cells and myocardium was examined by transmission electron microscopy at 2 h after transplantation.

### Establishment of MI Model and Cell Transplantation

Thirty-two adult female SD rats (200 ± 20 g) were anesthetized with ketamine (80 mg/kg) and xylazine (3 mg/kg). After endotracheal intubation, the heart was exposed, and the left anterior descending coronary artery (LAD) was ligated [30]. In sham group (n = 5), the needle passed through the ventricular wall around LAD without ligation. At 1 week after LAD ligation, two rats died of heart failure. The rest rats were divided into control (n = 6), c-kit^+^VEGFR-2^−^ MSCs (n = 6), c-kit^+^VEGFR-2^+^ MSCs (n = 6) and precondition (n = 7) groups. In control group, 40 μl fibrinogen (10 mg/ml) and 40 μl thrombin (10 IU/ml) were injected simultaneously with a Duploject syringe into the peri-infarcted region at four points. In the cell or precondition (c-kit^+^VEGFR-2^+^ MSCs pretreated with 1% O_2_ and 3% FBS for 4 h) groups, 1 × 10^6^ cells were suspended in fibrinogen before injection. Before transplantation, the cells were transfected with GFP (green fluorescent protein) gene.

### Echocardiography

Echocardiograms were recorded with Vevo 2100 Imaging System (Visual Sonics, Toronto, ON, Canada) before MI, at 1 week after MI (before transplantation) and 4 weeks after transplantation respectively. The rats were anesthetized with isoflurane and fixed on the metal plate for detection. After adequate two-dimensional images were obtained, the M-mode cursor was set to the parasternal long axis at the level of the papillary muscles. The LV end-diastolic diameter (LVEDD) and LV end-systolic diameter (LVESD) were measured from at least three consecutive cardiac cycles. To evaluate the systolic function, the LV end-diastolic volume (LVEDV), the LV end-systolic volume (LVESV), the ejection fraction (EF = LVEDV-LVESV/LVEDV × 100%) and fractional shortening (FS = LVEDD − LVESD/LVEDD × 100%) were examined. Successful establishment of MI model was confirmed by EF of < 50% and FS of < 30%. Six measurements at least were taken and averaged for each parameter.

### Masson’s Trichrome Staining

To evaluate myocardial repair of the infarcted region, the cryosections were stained with Masson’s trichrome. At 4 weeks after transplantation, the hearts were removed and perfused with 4% paraformaldehyde solution. Then, the hearts were cut into upper and lower parts along the cross-section, and continued to be fixed with 4% paraformaldehyde solution overnight. After washing with DPBS, the tissue was treated with 15% and 30% sucrose gradient to dehydration, and then embedded with Tissue-Tek OCT (Sakura Finetek, Torrance, CA, USA). The cryosections (5-μm thickness) were prepared. Scar tissue with plentiful collagen was stained blue, while myocardial tissue was stained red. The scar area was defined as percentage of circumference of the infarct region in the whole LV wall circumference, and measured by Image J 1.46r (National Institutes of Health, Bethesda, MD, USA). The thickness of the LV wall at the infarcted region was measured at the thinnest part of the region. At least 5 independent sections and 2 fields (20 x) on each section were selected randomly.

### Immunostaining of the Myocardium

Angiogenesis and myocardial regeneration of the infarcted region were examined with immunostaining. The cryosections were incubated with mouse anti-rat CD31 antibody (1:200) overnight, and then incubated with Alexa Fluor 594-conjugated goat anti-mouse IgG (1:400) for 30 min. Moreover, the cryosections were incubated with mouse anti-rat cTnT antibody (1:200) and rabbit anti-rat Cx43 (connexin-43) antibody (1:100; Abcam, Cambridge, MA, USA) overnight, followed by incubation with Alexa Fluor 594-conjugated goat anti-mouse IgG and Alexa Fluor 488-conjugated goat anti-rabbit IgG (1:400) for 30 min.

To trace differentiation of the transplanted cells into endothelial cells, smooth muscle cells and cardiomyocytes, double immunostaining of GFP and CD31, α-SMA or cTnT was performed. The cryosections were incubated with rabbit anti-rat GFP antibody (1:100; Santa Cruz Biotechnology) and mouse anti-rat CD31 antibody (1:200), mouse anti-rat α-SMA antibody (1:200; Abcam) or mouse anti-rat cTnT antibody (1:200) respectively. The gap junction between cardiomyocyte differentiated from the transplanted cell and resident cardiomyocyte was identified by triple immunostaining of GFP, cTnT and Cx43. The cryosections were incubated with chicken anti-rat GFP antibody (1:200; Novus), mouse anti-rat cTnT antibody (1:200) and rabbit anti-rat Cx43 antibody (1:100) overnight, followed by incubation with DyLight 488-conjugated goat anti-chicken lgG (1:200; Novus), Alexa Fluor 594-conjugated goat anti-mouse IgG (1:400; Abcam) and Alexa Fluor 647-conjugated goat anti-rabbit IgG (1:400; Jackson ImmunoResearch) for 30 min.

### Statistical Analysis

Data were presented as means ± standard deviation in the experiments above. Statistical analysis was conducted by SPSS 17.0 software (SPSS, Chicago, IL, USA) using t-test and one-way ANNOVA. *p* <  0.05 was regarded as the statistically significance.

## Results

### Phenotype and Gene Expression of c-kit^+^VEGFR-2^+^ MSCs

**Fig. 1:**
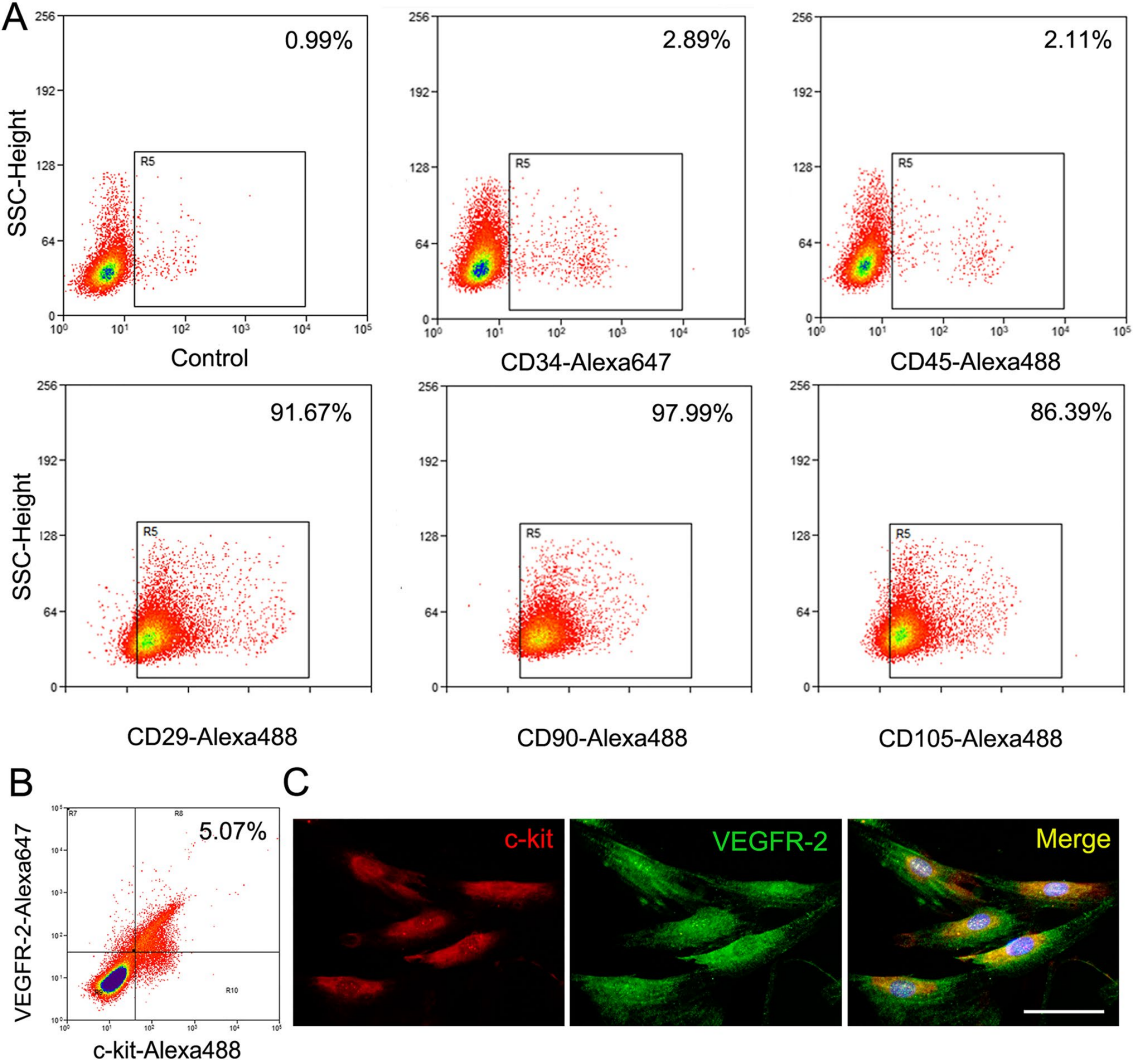
Characterization of c-kit^+^VEGFR-2^+^ MSCs. **A** The flow cytometric analysis of CD34^−^, CD45^−^, CD29^+^, CD90^+^ or CD105^+^ cells in MSCs isolated from the mononuclear cells of rat bone marrow. **B** The phenotype of MSCs analyzed by dual-color flow cytometry. Percentage of the positive cells was compared to isotype control. **C** The sorted c-kit^+^VEGFR-2^+^ MSCs. Immunostaining. Scale bar = 50 μm

There was a population of c-kit^+^VEGFR-2^+^ cells in MSCs isolated from bone marrow. MSCs expressed mesenchymal lineage markers (CD29, CD90 and CD105), and were negative for hematopoietic markers (CD34 and CD45) (Fig. 1A). The frequency of c-kit^+^VEGFR-2^+^ cells sorted from MSCs was 5.07 ± 0.40% (Fig. 1B), about 70% of c-kit^+^ cells. Figure 1C showed immunostaining of c-kit^+^VEGFR-2^+^ MSCs.

**Fig. 2:**
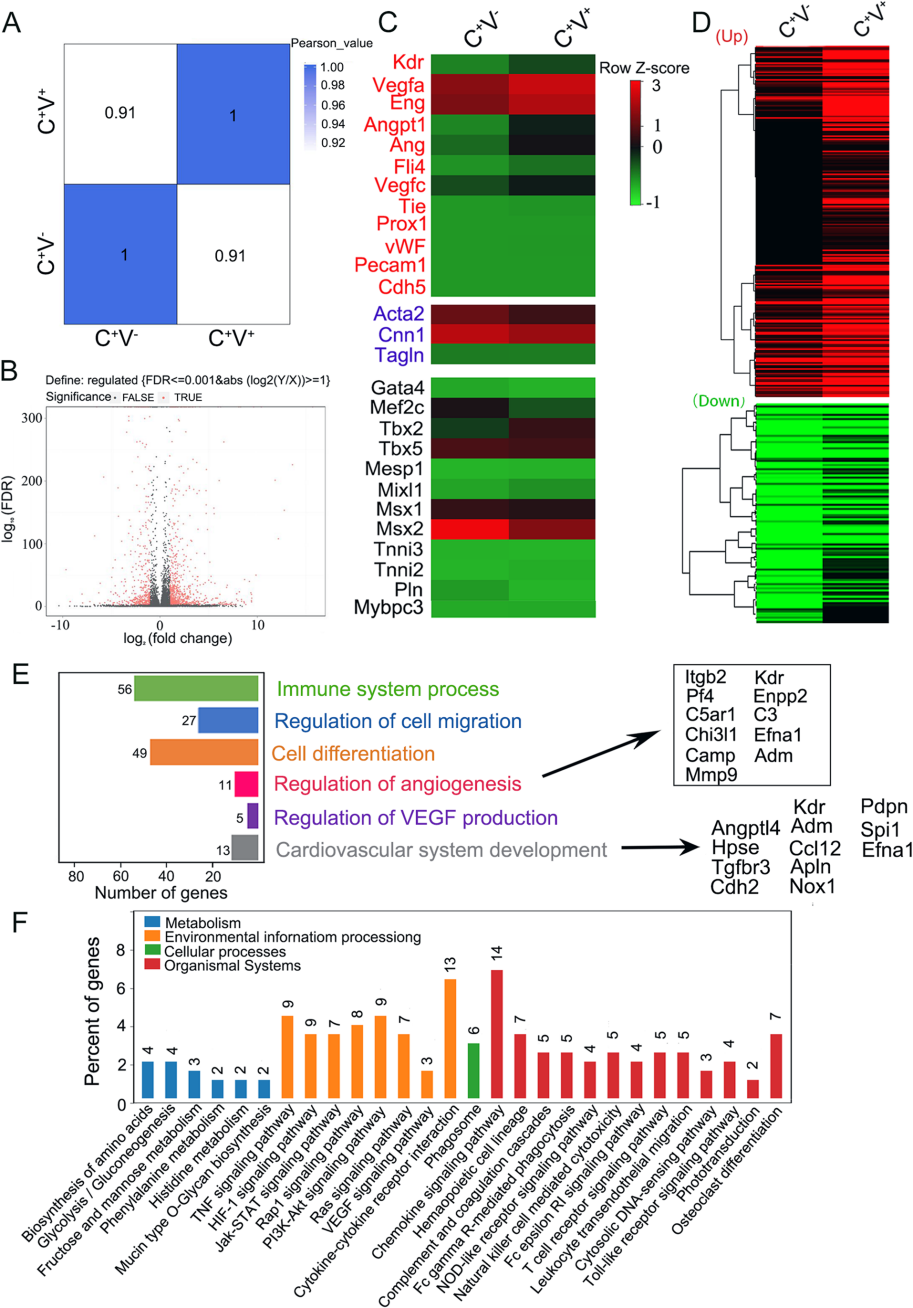
Gene expression profile of c-kit^+^VEGFR-2^+^ MSCs. **A** Pearson’s correlation coefficient of C^+^V^−^ and C^+^V^+^ groups. C^+^V^−^, c-kit^+^VEGFR-2^−^ MSCs; C^+^V^+^, c-kit^+^VEGFR-2^+^ MSCs. **B** Volcano plot of all expressed genes. Compared with expression of the genes in c-kit^+^VEGFR2^−^ MSCs, c-kit^+^VEGFR2^+^ MSCs contained 707 upregulated genes and 414 downregulated genes. **C** Dynamic expression of cardiovascular lineage genes. Types of markers were color-coded in heat map. Red, endothelial cell markers; blue, smooth muscle cell markers; black, cardiomyocyte markers. Expression of most endothelium-specific genes such as *Kdr*, *Vegfa*, *Angpt1*, *Ang*, *Flt4*, *Vegfc*, *Tie* and *Eng* in c-kit^+^VEGFR2^+^ MSCs was higher than that in c-kit^+^VEGFR2^−^ MSCs. **D** Hierarchical clustering of the significantly unregulated genes and downregulated genes. **E** Gene ontology analysis of the upregulated and differential genes. The genes related to angiogenesis and cardiovascular system development are overrepresented significantly in c-kit^+^VEGFR-2^+^ MSCs. **F** KEGG pathway analysis of biological processes based on the upregulated genes. The upregulated genes are enriched in VEGF, HIF and chemokine signaling pathways, which shows the superiority of the c-kit^+^VEGFR2^+^ MSCs in cytokine secretion and vascular formation

Gene expression profile revealed that there were 12,491 genes in c-kit^+^VEGFR2^+^ MSCs and 12,231 genes in c-kit^+^VEGFR2^−^ MSCs, which counted 71.59% and 70.10% as total gene numbers in Database respectively. Pearson’s correlation coefficient values acrossing two samples was 0.91, which indicated a high consistence of whole genes in two samples (Fig. 2A). The volcano plot of all expressed genes was shown in Fig. 2B. Compared with expression of the genes in c-kit^+^VEGFR2^−^ MSCs, c-kit^+^VEGFR2^+^ MSCs contained 707 upregulated genes and 414 downregulated genes. Expression of most endothelium-specific genes (*Kdr*, *Vegfa*, *Angpt1*, *Ang*, *Flt4*, *Vegfc*, *Tie* and *Eng*) in c-kit^+^VEGFR2^+^ MSCs was higher than that in c-kit^+^VEGFR2^−^ MSCs, while expression of most smooth muscle-specific and myocardium-specific genes showed no significant difference between two populations of cells. Expression of mature endothelium-specific genes such as *Pecam1* and *vWF* was minimally observed in c-kit^+^VEGFR2^+^ MSCs as well as c-kit^+^VEGFR2^−^ MSCs, which coincided with stemness of MSCs (Fig. 2C). The results of RT-PCR showed that the expression levels of KDR, VEGFA, Angpt1 and Ang in c-kit^+^VEGFR-2^+^ MSCs were higher than that in c-kit^+^VEGFR-2^−^ MSCs significantly. However, the differences in expression of Flt4, VEGFC, Tbx2 and Tbx5 in two phenotypes of cells were not significant (Fig. S1). The 232 upregulated genes and 104 downregulated genes of c-kit^+^VEGFR-2^+^ MSCs (log_2_FC > 1.5 or Log_2_FC < -1.5 in all three experiments) were clustered respectively (Fig. 2D). Gene ontology analysis of the upregulated genes illustrated enrichment of genes related to immune process, cell migration, cell differentiation, angiogenesis (*Itgb2*, *Pf4*, *C5ar1*, *Chi3l1*, *Camp*, *Mmp9*, *Kdr*, *Enpp2*, *C3*, *Efna1* and *Adm*), VEGF production and cardiovascular system development (*Angpt1*, *Angptl4*, *Hpse*, *Tgfbr3*, *Cdh2*, *Nox1*, *Spil*, *Kdr*, *Adm*, *Ccl12*, *Pdpn*, *Apln*, and *Efna1*). Some typical pathways reflecting cell function such as blood vessel morphogenesis and cytokine production were overrepresented significantly in c-kit^+^VEGFR-2^+^ MSCs (Fig. 2E), and their first neighbor network construction was demonstrated in Fig. S2. KEGG pathway analysis confirmed that the upregulated genes were enriched in VEGF, HIF and chemokine signaling pathways, which showed the superiority of the cells in cytokine secretion and vascular formation. Moreover, in terms of metabolism, the upregulated genes were also enriched in biosynthesis of amino acids and glycolysis, which indicated that c-kit^+^VEGFR2^+^ MSCs possess high tolerance to hypoxia than c-kit^+^VEGFR2^−^ MSCs (Fig. 2F).

### Properties of c-kit^+^VEGFR-2^+^ MSCs

**Fig. 3:**
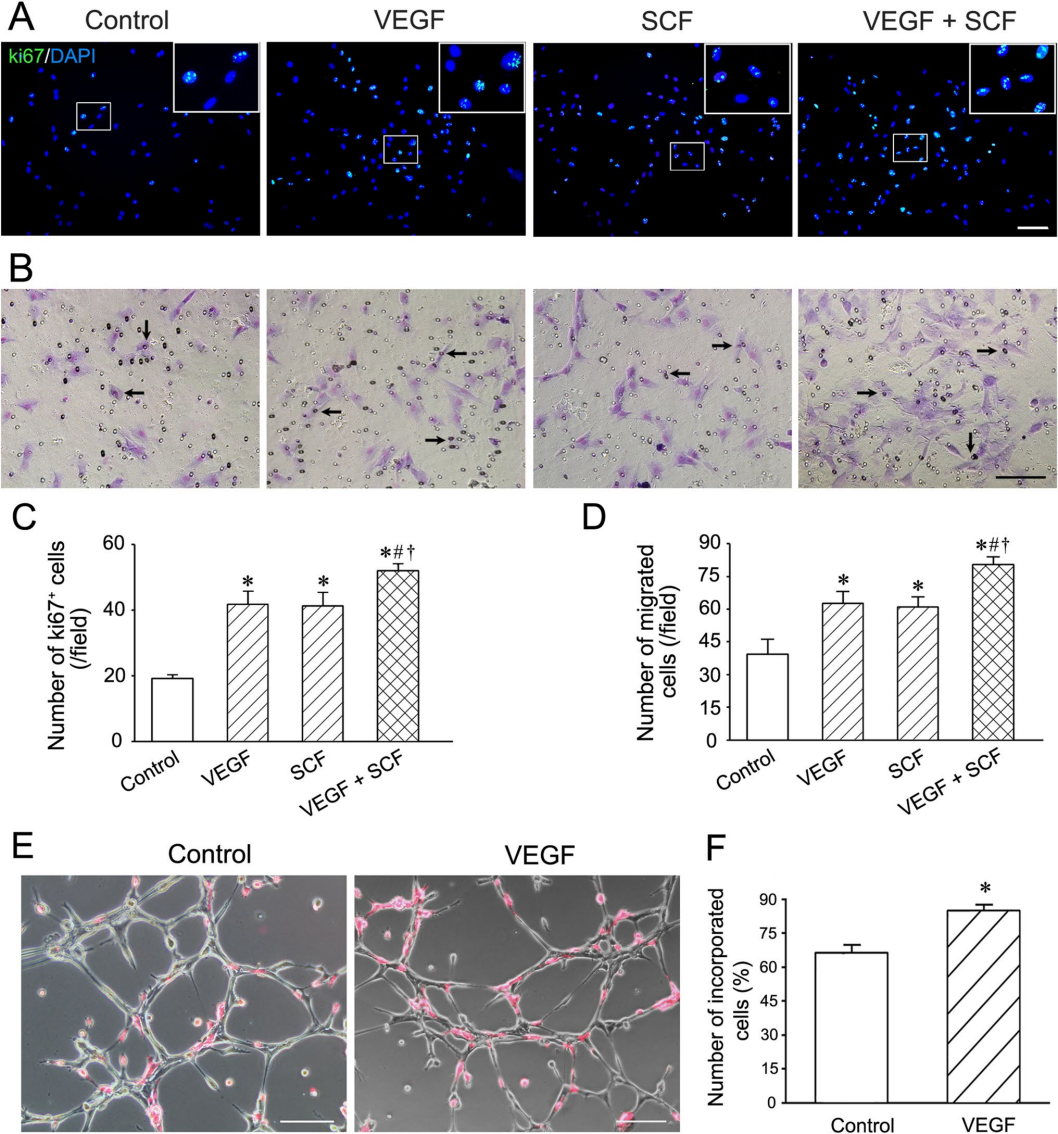
Proliferation, migration and incorporation of c-kit^+^VEGFR-2^+^ MSCs. **A** Effects of VEGF and SCF on proliferation of the cells. The cells were treated with the growth factors for 24 h. The large boxes are magnification of the small boxes. Ki-67 immunostaining. **B** Effects of VEGF and SCF on migration of the cells. The cells were treated with the growth factors for 12 h. Arrows indicate the cells passing through the pores of the membrane. Scale bar = 100 μm. **C** Statistical result of the number of Ki-67^+^ cells. **D** Statistical result of the number of the migrated cells. *p < 0.05 versus control group, #*p* < 0.05 versus VEGF group, †*p* < 0.05 versus SCF group. n = 6. **E** Incorporation of c-kit^+^VEGFR-2^+^ MSCs to the capillary-like structures formed by pulmonary microvascular endothelial cells. c-kit^+^VEGFR-2^+^ MSCs were labelled with Dil. Scar bar = 100 μm. **F** The statistical result of the incorporated c-kit^+^VEGFR-2^+^ MSCs after VEGF induction. *p < 0.05 versus control group. n = 4

c-kit^+^VEGFR-2^+^ MSCs possessed properties of proliferation, migration and formation of capillary-like structures in three-dimensional Matrigel. After treatment with VEGF and SCF, the proliferated cells and migrated cells were increased significantly. The numbers of the proliferated cells and migrated cells in VEGF + SCF group were greater than VEGF group or SCF group (Fig. 3A–D). Compared with the control group, more c-kit^+^VEGFR2^+^ MSCs incorporated to the capillary-like structures formed by the endothelial cells in VEGF group (Fig. 3E, F).

**Fig. 4:**
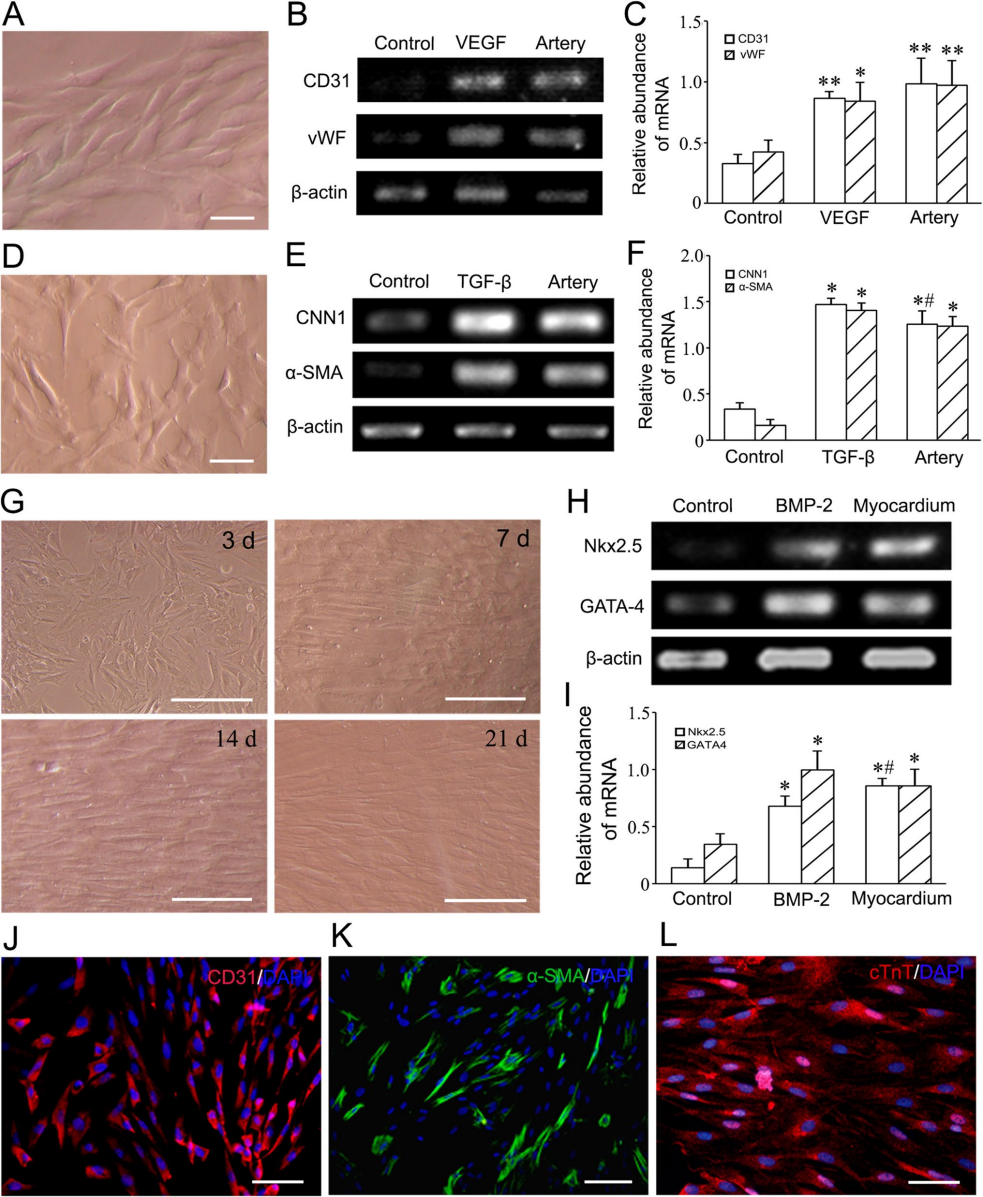
Differentiation of c-kit^+^VEGFR-2^+^ MSCs into cardiovascular cells after induction. **A** The phase contrast image of VEGF-induced cells. Scale bar = 50 μm. **B** Expression of CD31 and vWF mRNAs in the VEGF-induced cells. **C** Statistical result of expression of CD31 and vWF mRNAs. The femoral artery from SD rat as positive control. *p < 0.05, **p < 0.01 versus control group. n = 3. **D** The phase contrast image of TGF-β-induced cells. Scale bar = 50 μm. **E** Expression of CNN1 and α-SMA mRNAs in the TGF-β-induced cells. **F** The statistical result of expression of CNN1 and α-SMA mRNAs. The femoral artery from SD rat as positive control. *p < 0.05 versus control group, #*p* < 0.05 versus TGF-β group. n = 3. **G** The phase contrast images of the cells at 3, 7, 14 and 21 days after induction with BMP-2. Scale bar = 100 μm. **H** Expression of Nkx2.5 and GATA4 mRNAs in the BMP-2-induced cells. **I** The statistical result of expression of Nkx2.5 and GATA4 mRNAs. The myocardium from SD rat as positive control. *p < 0.05 versus control group, #*p* < 0.05 versus BMP-2 group. n = 3. **J**–**L** Expression of CD31, α-SMA and cTnT in the differentiated cells. Immunostaining. Scale bar = 50 μm. In **A**–**F** and **H–K**, the cells were induced for 2 weeks. In **G** and **L**, the cells were induced for 3 weeks

The results of RT-PCR and immunostaining demonstrated that c-kit^+^VEGFR2^+^ MSCs could differentiate into endothelial cells, smooth muscle cells and myocardiocytes after induction. After induction with VEGF for 2 weeks, the cells displayed a typical cobblestone appearance like endothelial cells, and expressed *CD31* and *vWF* (Fig. 4A–C). At 2 weeks after induction with TGF-β, most cells represented long spindle shape like smooth muscle cells. The cells expressed *CNN1* and *α-SMA* (Fig. 4D–F). After induction with BMP-2, the cells tended to parallel alignment (Fig. 4G). At 2 weeks after induction, the cells expressed *NKX2.5* and *GATA-4* (Fig. 4H, I). In immunostaining, the induced cells were positive for CD31 (vascular endothelial cell maker), α-SMA (smooth muscle cell marker) and cTnT (cardiomyocyte marker) respectively (Fig. 4J–L).

### Optimal Preconditioning of Hypoxia and Serum Deprivation

**Fig. 5:**
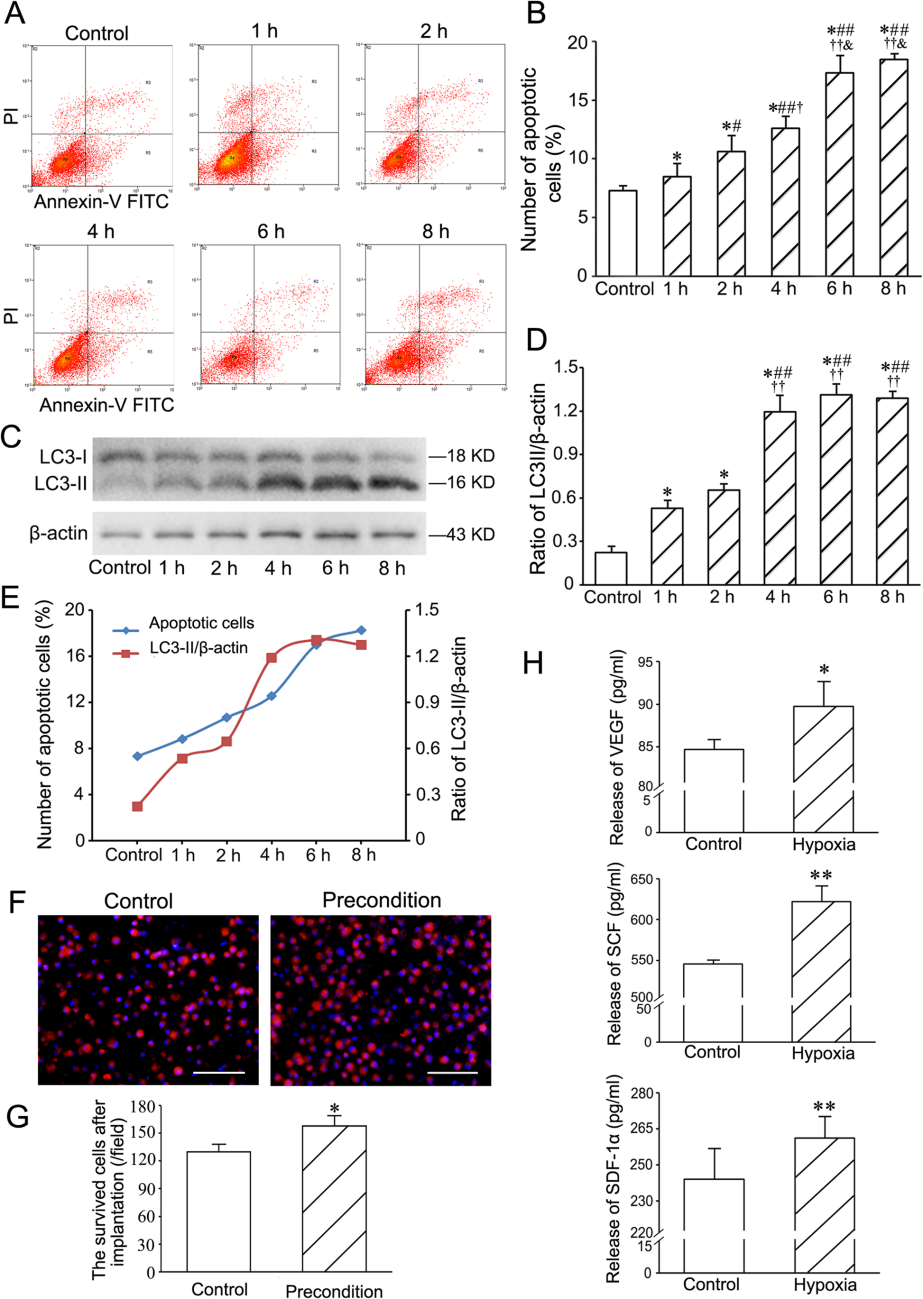
Determination of the optimal preconditioning of hypoxia and serum deprivation for c-kit^+^VEGFR-2^+^ MSCs. **A** The typical quadrantal diagrams of flow cytometric analysis of the apoptotic cells. The cells were treated with 1% O_2_ and 3% FBS. **B** The statistic result of the numbers of the apoptotic cells. *p < 0.05 versus control group, #*p* < 0.05 and ##*p* < 0.01 versus 1 h group, †*p* < 0.05 and ††*p* < 0.01 versus 2 h group, & *p* < 0.05 versus 4 h group. n = 3. **C** Expression of LC3 in the cells. Western blotting. **D** The statistic result of LC3II/β-actin ratios. *p < 0.05 versus control group, ##*p* < 0.01 versus 1 h group, ††*p* < 0.01 versus 2 h group. n = 4. **E** The curves of the numbers of the apoptotic cells and LC3-II/β-actin ratios. **F** The survived cells in the cells preconditioned with hypoxia and serum deprivation for 4 h after implantation into the abdominal ischaemic pouch for 24 h. Scale bar = 100 μm. **G** The statistical result of the numbers of the survived cells. *p < 0.05 versus control group. n = 6. **H** Concentration of VEGF, SCF and SDF-1α from the cells treated with hypoxia (1% O_2_) and serum deprivation (3% FBS) for 12 h. *p < 0.05, **p < 0.01 versus control group. n = 6

After treatment with hypoxia (1% O_2_) and serum deprivation (3% FBS), the number of the apoptotic cells constantly increased. Ratio of LC3-II/β-actin reached the plateau at 4 h after treatment. According to the balance of autophagy and apoptosis, the level of autophagy was near to the plateau at 4 h after treatment, while the degree of apoptosis was not much high at this time point (Fig. 5A–E). Therefore, 4 h was regarded as an optimal preconditioning time for c-kit^+^VEGFR-2^+^ MSC transplantation. In the abdominal ischaemic pouch assay, the number of the survived cells in the cells preconditioned with hypoxia and serum deprivation for 4 h was greater than control group (Fig. 5F, G). This result shows that preconditioning is effective for improving survival of the transplanted cells.

After treatment with hypoxia and serum deprivation for 12 h, concentrations of VEGF, SCF and SDF-1α secreted from c-kit^+^VEGFR-2^+^ MSCs were increased significantly (Fig. 5H).

### Compatibility of Fibrin with the Implanted Cells and Myocardium

In the images of scanning electron microscope, fibrin constituted a delicate fibrous network, and the cells spread well on the network (Fig. S3A, B). In the images of transmission electron microscope, the cells grew well in the fibrin gel (Fig. S3C). Fibrin represents a good compatibility with the implanted cells and myocardium (Fig. S3D, E).

### Improvement of Cardiac Function after Cell Transplantation

**Fig. 6:**
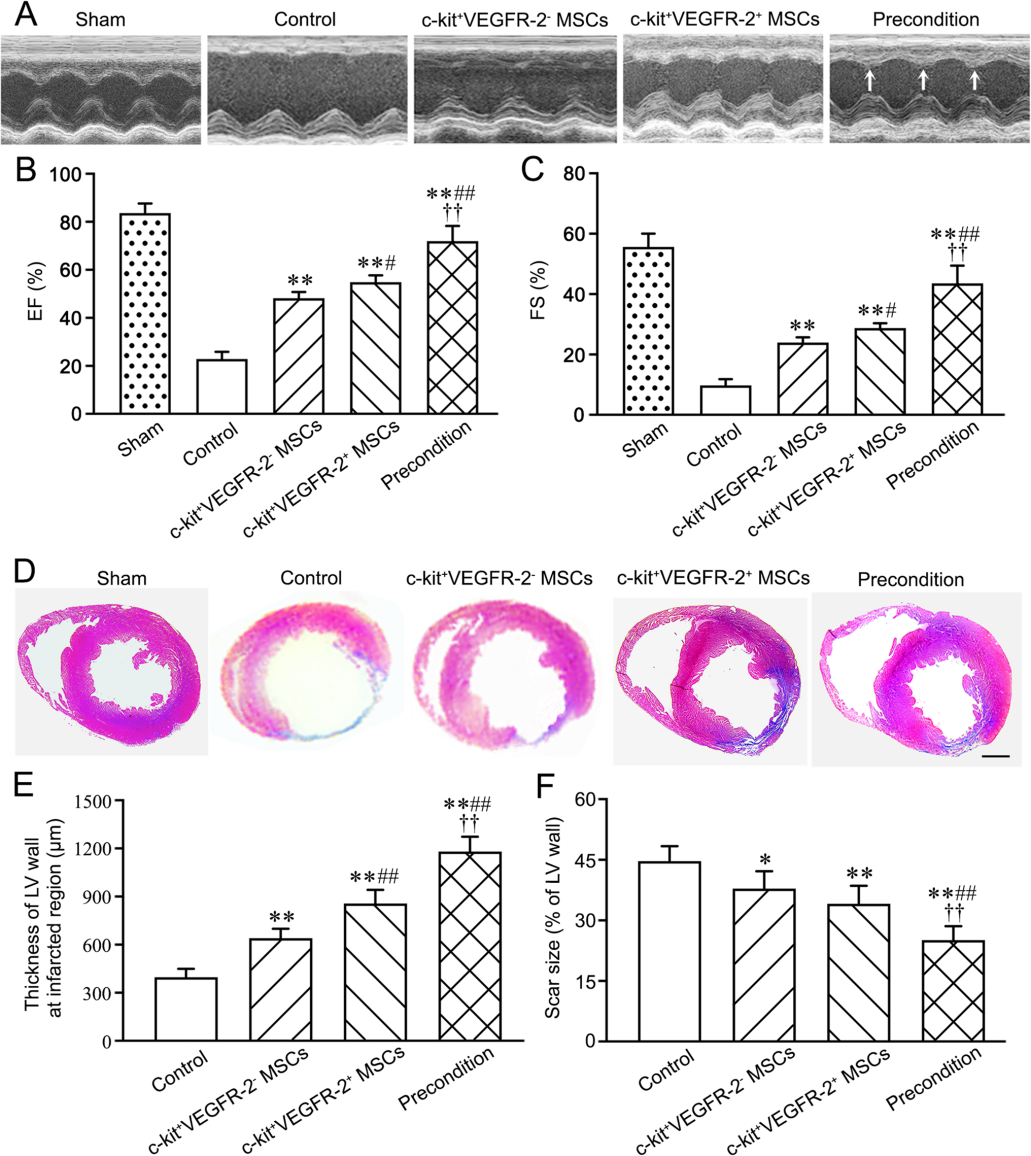
Changes of cardiac function and structure at 4 weeks after cell transplantation. **A** Representative echocardiograms of LV free walls. LV contraction in the precondition group is improved significantly. Arrows indicate the contraction waves of LV anterior wall. **B**, **C** The statistical results of EF and FS of the left ventricles. **p < 0.01 versus control group; #*p* < 0.05, ##*p* < 0.01 versus c-kit^+^VEGFR-2^−^ MSC group; ††*p* < 0.01 versus c-kit^+^VEGFR-2^+^ MSC group. **D** The transverse sections of the ventricles at the widest part of the infarct region. There is more myocardium (red) and less fibrous tissue (blue) in precondition group than c-kit^+^VEGFR-2^+^ MSC group. Masson’s trichrome staining. Scale bar = 2 mm. **E**, **F** The statistical results of the thickness of LV wall at the thinnest part of the infarct region and scar size. *p < 0.05, **p < 0.01 versus control group; ##*p* < 0.01 versus c-kit^+^VEGFR-2^−^ MSC group; ††*p* < 0.01 versus c-kit^+^VEGFR-2^+^ MSC group. n = 6

Representative echocardiograms of the LV free walls were shown in Fig. 6A. The echocardiograms revealed that cardiac function in all rats was severely compromised at 1 week post-MI. In the control group, the loss of cardiac function lasted for 4 weeks. At 4 weeks after transplantation, LV contraction in c-kit^+^VEGFR-2^−^ MSC group or c-kit^+^VEGFR-2^+^ MSC group was significantly improved, while LV contraction in c-kit^+^VEGFR-2^+^ MSC group was stronger than c-kit^+^VEGFR-2^−^ MSC group. Compared with c-kit^+^VEGFR-2^+^ MSC group, LV contraction in precondition group was strengthened significantly. EF and FS in c-kit^+^VEGFR-2^+^ MSC group were greater than c-kit^+^VEGFR-2^−^ MSC group. Compared with c-kit^+^VEGFR-2^+^ MSC group, EF and FS in precondition group were increased remarkably (Fig. 6B, C).

### Histological Changes of the LV Wall after Transplantation

The morphological changes of the LV wall were shown in Fig. 6D. In the control group, the myocardium of the infarcted region was replaced by fibrous tissue at 4 weeks post-MI. There was more myocardium at the infarcted region after cell transplantation for 4 weeks. The myocardium in c-kit^+^VEGFR-2^+^ MSC group was more than c-kit^+^VEGFR-2^−^ MSC group. Compared with c-kit^+^VEGFR-2^+^ MSC group, repair of the myocardium in precondition group was increased significantly. The thickness of LV wall at the infarcted region after cell transplantation was increased, while difference in the thickness between c-kit^+^VEGFR-2^+^ MSC group and c-kit^+^VEGFR-2^−^ MSC group was significant. The thickness of the LV wall in precondition group was greater than c-kit^+^VEGFR-2^+^ MSC group (Fig. 6E). The scar size was decreased after cell transplantation. Compared with c-kit^+^VEGFR-2^+^ MSC group, the scar size in precondition group was decreased significantly (Fig. 6F).

### Angiogenesis and Myocardium Regeneration after Transplantation

**Fig. 7:**
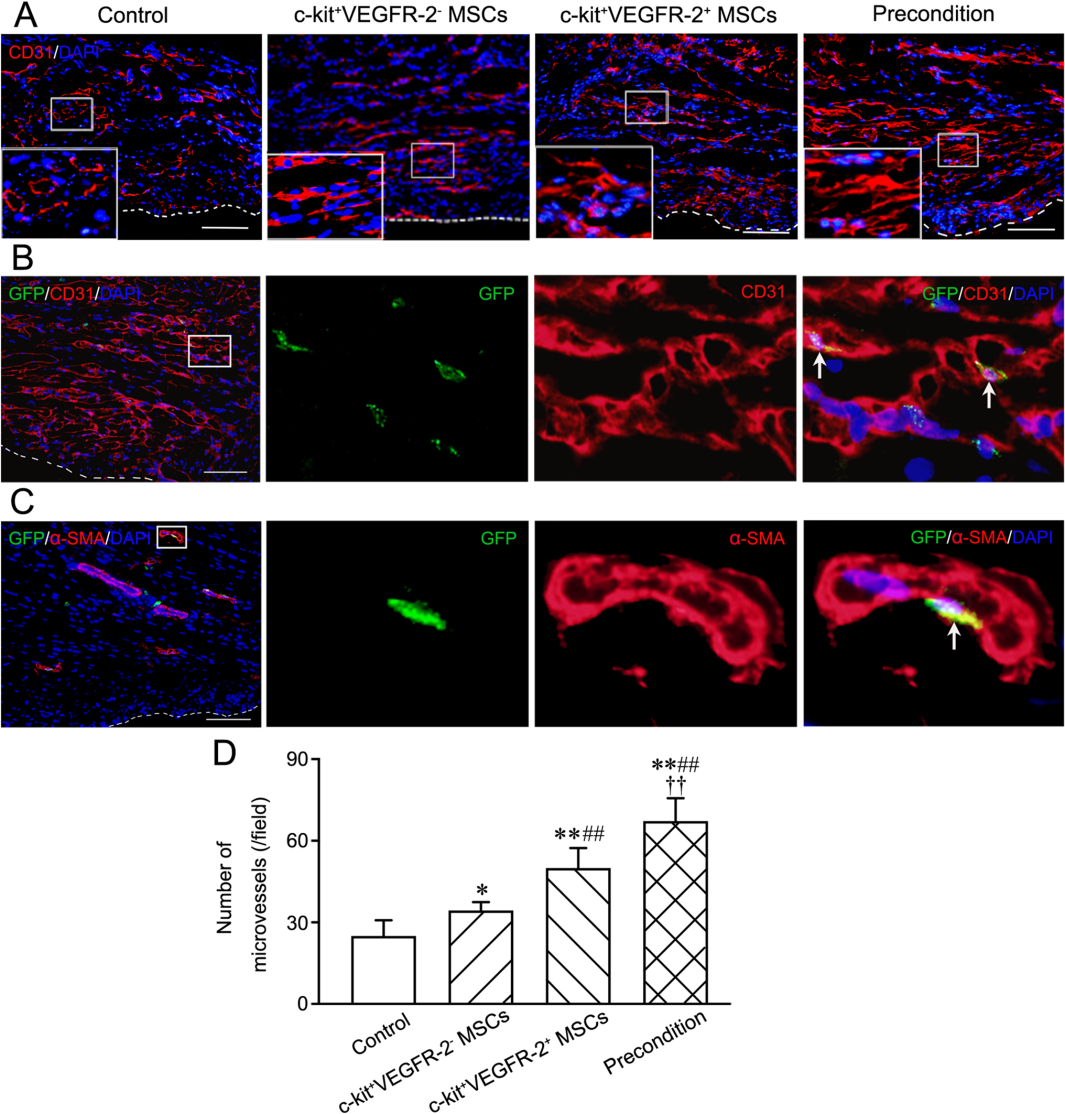
Angiogenesis and differentiation of the engrafted cells into vascular cells at infarcted region at 4 weeks after transplantation. **A** The regenerated microvessels. The large boxes are magnification of the small boxes. The white dash line indicates the surface of the epicardium. **B**, **C** The endothelial cells (GFP^+^CD31^+^ cells) and smooth muscle cells (GFP^+^α-SMA^+^ cells) differentiated from the engrafted c-kit^+^VEGFR-2^+^ MSCs in precondition group. The panels from the second to forth column are magnification of the boxes in the panels of the first column. The arrows indicate the differentiated cells incorporated into the wall of the microvessels. Immunostaining. Scale bar = 100 μm. **D** The statistical result of the numbers of the microvessels. *p < 0.05, **p < 0.01 versus control group; ##*p* < 0.01 versus c-kit^+^VEGFR-2^−^ MSC group; ††*p* < 0.01 versus c-kit^+^VEGFR-2^+^ MSC group. n = 6

After transplantation for 4 weeks, angiogenesis in infarcted region was assessed by counting CD31^+^ microvessels. The regenerated microvessels were increased after cell transplantation. The microvessels in c-kit^+^VEGFR-2^+^ MSC group were more than c-kit^+^VEGFR-2^−^ MSC group. Compared with c-kit^+^VEGFR-2^+^ MSC group, the number of the microvessels in precondition group was greater significantly (Fig. 7A, D). In precondition group, some engrafted c-kit^+^VEGFR-2^+^ MSCs differentiated into endothelial cells (GFP^+^CD31^+^ cells) and smooth muscle cells (GFP^+^α-SMA^+^ cells). The differentiated cells were incorporated into the wall of the microvessels (Fig. 7B, C).

**Fig. 8:**
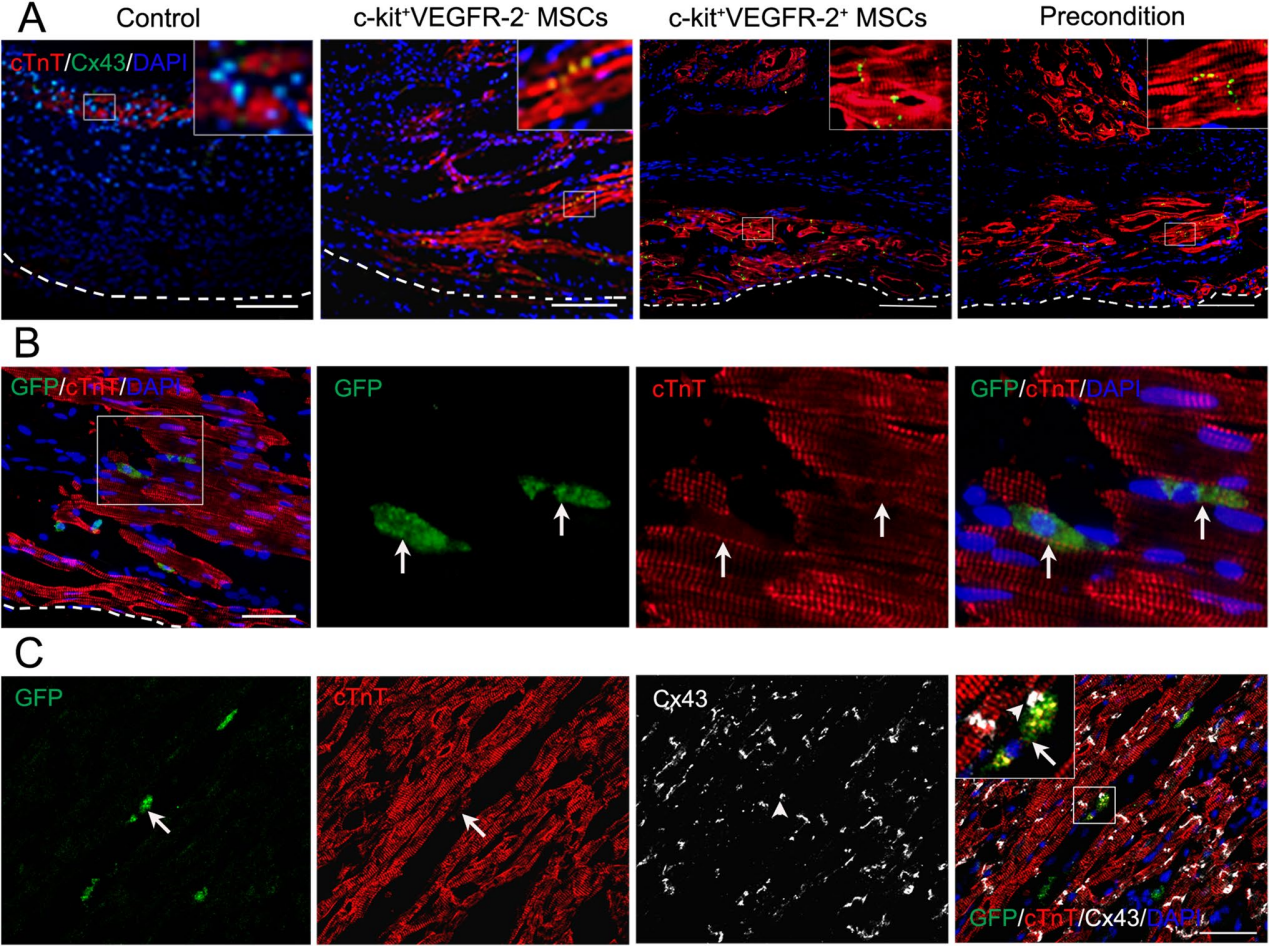
Myocardium regeneration and differentiation of the engrafted cells into cardiomyocytes at infarcted region at 4 weeks after transplantation. **A** The regenerated myocardium. The large boxes are magnification of the small boxes. The white dash line indicates the surface of the epicardium. **B** The cardiomyocytes (GFP^+^cTnT^+^ cells) differentiated from the engrafted c-kit^+^VEGFR-2^+^ MSCs in precondition group. The arrows indicate the differentiated cells. **C** Cx43 expression in precondition group. Cx-43 is located between the cardiomyocyte differentiated from c-kit^+^VEGFR-2^+^ MSC and native cardiomyocyte. The large box is magnification of the small box. The arrow indicates the differentiated cell. The arrowhead indicates location of Cx43. Immunostaining. Scale bar = 100 μm (**A**, **C**), 50 μm (**B**)

Degree of myocardial regeneration at the infarcted region at 4 weeks after transplantation in each group in Fig. 8A was similar to that in Fig. 6D. Subepicardial myocardial regeneration is prominent in cell transplantation groups especially in c-kit^+^VEGFR-2^+^ MSC group and precondition group. In precondition group, some engrafted c-kit^+^VEGFR-2^+^ MSCs differentiated into cardiomyocytes (GFP^+^cTnT^+^ cells). The cardiomyocytes differentiated from the engrafted cells were parallel to native cardiomyocytes (Fig. 8B). The result of triple immunostaining of GFP, cTnT and Cx43 in precondition group showed that Cx-43 was located between the cardiomyocyte differentiated from c-kit^+^VEGFR-2^+^ MSC and native cardiomyocyte (Fig. 8C).

### Enhancement of Paracrine in the Peri-Infarcted Myocardium after Transplantation

At 1 week after transplantation, concentrations of VEGF, SCF and SDF-1α in plasma were higher in precondition group than c-kit^+^VEGFR-2^+^ MSC group. Compared with the control group, concentration of SDF-1α in the c-kit^+^VEGFR-2^+^ MSC group was higher. In the peri-infarcted myocardium, concentrations of these cytokines in the c-kit^+^VEGFR-2^+^ MSC group were higher than that in the control group. Compared with the c-kit^+^VEGFR-2^+^ MSC group, concentrations of these cytokines in the precondition group were increased significantly (Fig. S4).

## Discussion

In this study, we found that there is population of c-kit^+^VEGFR-2^+^ cells in bone marrow-derived MSCs. The number of the VEGFR-2^+^ cells is 70% in c-kit^+^ MSCs. This study highlights the heterogeneity of c-kit MSCs. c-kit^+^VEGFR-2^+^ MSCs have a potential to differentiate towards endothelial cells, smooth muscle cells and cardiomyocytes. c-kit^+^VEGFR-2^+^ MSCs express most smooth muscle-specific and myocardium-specific genes, while endothelium-specific genes are highly expressed especially ones related with angiogenesis. Compared with c-kit^+^VEGFR-2^−^ MSCs, the upregulated differential genes in c-kit^+^VEGFR-2^+^ MSCs are involved in cell migration, cell differentiation, and angiogenesis. Transplantation of c-kit^+^VEGFR-2^+^ MSCs is more effective in improving cardiac function and alleviating adverse ventricular remodeling post-MI. The engrafted c-kit^+^VEGFR-2^+^ MSCs can incorporate to the microvessels for participating in angiogenesis. Moreover, the differentiated cells can conjunct with native cardiomyocytes to form the functional myocardium especially the preconditioned cells. These results suggest that c-kit^+^VEGFR-2^+^ MSCs are a novel population of stem cells for MI therapy. This study and other studies [31] show that VEGFR-2 signalling plays a vital role in differentiation of stem/progenitor cells into endothelial cells. Therefore, transplantation of c-kit^+^VEGFR-2^+^ MSCs may be applied to promote angiogenesis in other ischaemic diseases such as critical limb ischaemia in diabetes.

The results of this study demonstrate that enhanced paracrine of c-kit^+^VEGFR-2^+^ MSCs and the myocardium after transplantation favours to engraftment and differentiation of c-kit^+^VEGFR-2^+^ MSCs in the ischaemic myocardium. In recent years, attention has been paid on paracrine mechanisms of reparative and regenerative effects of stem cells [32]. Clinical trials have suggested that secretion of paracrine factors may be the underlying mechanism responsible for the improvement in outcomes [33]. Both VEGF and SCF promote proliferation and migration of c-kit^+^VEGFR-2^+^ MSCs, while the effects of VEGF and SCF are synergetic. VEGF activates incorporation of c-kit^+^VEGFR-2^+^ MSCs to the microvessels. Moreover, SCF may induce recruitment and expansion of c-kit^+^ cells [34]. Expression of c-kit in CSCs is upregulated in response to pathological stress. Activation of c-kit receptor mediates cell survival and proliferation of stem cells [35]. Taken together, SCF/c-kit and VEGF/VEGFR-2 signalling pathways regulate the activities of c-kit^+^VEGFR-2^+^ MSCs. In addition, SDF-1α-CXCR4 axis plays a crucial role in homing of stem or progenitor cells from bone marrow to ischaemic myocardium [36]. MSCs can stimulate CSC chemotaxis via SDF-1α/CXCR4 signalling pathway [37]. The recent study shows that paracrine of the engrafted MSCs account for activation the epicardium and recruitment of endogenous stem cells [30]. Thus, effects of paracrine of c-kit^+^VEGFR-2^+^ MSCs on endogenous repair of the infarct myocardium are deserved to be explored. In view of that microvesicles play important roles in cell–cell communication, cell differentiation and organ regeneration [38], contribution of c-kit^+^VEGFR-2^+^ MSC-released microvesicles to repair of the infarcted myocardium after cell transplantation needs further investigation.

Survival and differentiation of the engrafted cells within hostile ischaemic and inflammatory microenvironment are poor [39]. Our experimental data reveal that preconditioning with hypoxia and serum deprivation may be beneficial for survival and differentiation of the transplanted c-kit^+^VEGFR-2^+^ MSCs. According to balance of autophagy and apoptosis induced with hypoxia and serum deprivation, treatment with 1% O_2_ and 3% FBS for 4 h was determined as optimal preconditioning of c-kit^+^VEGFR-2^+^ MSCs. Moderate rapamycin-primed MSCs can promote repair of the infarcted myocardium after transplantation [40]. Hypoxia-activated autophagy augments survival of endothelial progenitor cells by inhibiting apoptosis [41]. Hypoxia-preconditioned endothelial progenitor cells promote repair of the ischaemic hindlimb after transplantation [29]. Contribution of autophagy to preconditioning with hypoxia and serum deprivation enhances adaptation of the transplanted c-kit^+^VEGFR-2^+^ MSCs to ischaemic microenvironment.

We found that the fibrin gel protected the cells against apoptosis in the condition of hypoxia [29]. The results of this study showed that the fibrin gel represented good compatibility with the implanted cells and myocardium. Infarction of the myocardium leads to degradation of extracellular matrix, which influences the regenerative capability of the myocardium [42]. The fibrin gel may promotes retention and survival of the transplanted c-kit^+^VEGFR-2^+^ MSCs.

## Conclusions

This study suggests that bone marrow-derived c-kit^+^VEGFR-2^+^ MSCs have a potential to differentiate towards cardiovascular cells. The cells can effectively repair the infarcted myocardium after transplantation. Differentiation of c-kit^+^VEGFR-2^+^ MSCs as well as paracrine of the cells contributes to angiogenesis and myocardiac regeneration after transplantation. Moreover, this study provides a feasible approach to activate autophagy of the stem cells by preconditioning with hypoxia and serum deprivation. We propose that application of reliable stem cells and optimal strategies of transplantation may enhance efficiency of stem cell therapy for MI.

## Supplementary Information

Below is the link to the electronic supplementary material.

Supplementary file1 (DOC 4259 KB)

## Data Availability

The data and materials are available from the corresponding author on reasonable request.
