# Supplementary material for: c-kit+VEGFR-2+ Mesenchymal Stem Cells Differentiate into Cardiovascular Cells and Repair Infarcted Myocardium after Transplantation
Source: Stem Cell Rev Rep. 2022 Aug 13;19(1):230–47. doi: 10.1007/s12015-022-10430-z (PMC9823054; doi:10.1007/s12015-022-10430-z)
Supplement: Supplementary file 1 — Supplementary file1 (DOC 4259 KB) [file 12015_2022_10430_MOESM1_ESM.doc]

**SUPPLEMENTARY MATERIALS**

**c-kit^+^VEGFR-2^+^ mesenchymal stem cells differentiate into cardiovascular cells and repair infarcted myocardium after transplantation**

***Running head: Differentiation of c-kit^+^VEGFR-2^+^ MSCs***

Pei Zhou^1^, Shu-na Yu^1^, Hai-feng Zhang, Yong-li Wang, Ping Tao, Yu-zhen Tan*, Hai-jie Wang*

Department of Anatomy, Histology and Embryology, Shanghai Medical School of Fudan University, Shanghai 200032, China

To whom correspondence should be addressed to:

Hai-jie Wang Professor, MD., PhD.

Department of Anatomy, Histology and Embryology

Shanghai Medical School of Fudan University

138 Yixueyuan Road, Shanghai 200032, People’s Republic of China

Tel: +86-21-54237430

E-mail: hjwang@shmu.edu.cn

^1^ These authors contributed equally to this work.

* Corresponding author: Department of Anatomy, Histology and Embryology, Shanghai Medical School of Fudan University, 138 Yixueyuan Road, Shanghai 200032, People’s Republic of China.

E-mail: yztan@shmu.edu.cn (Y-Z Tan); hjwang@shmu.edu.cn (H-J Wang)

**Table S1 The sequences of the primers**

| Symbol | Version | Sequence (5’ to 3’) | Length |
| --- | --- | --- | --- |
| Ang | NM_001006992.1 | (F) 5′-GTCGGTTTTTGTGCTGGGTC-3′  (R) 5′-CCTTGATGCTGCCCTTGTTG-3′ | 209 bp |
| Angpt1 | NM_053546.2 | (F) 5′-GCTGGCAGTACAATGACAGT-3′  (R) 5′-GTATCTGGGCCATCTCCGAC-3′ | 358 bp |
| CD31 | NM_031591.1 | (F) 5′-AGGTGACCGTGGACAAAAAG-3′  (R) 5′-TGGCAGCGAAACACTAACAG-3′ | 217 bp |
| CNN1 | NM_031747.2 | (F) 5’-CCCACAATCACCACCCACACAACTA-3’  (R) 5’-TCATCTCCCCAAACTGTAACCCTAT-3’ | 164 bp |
| Flt4 | NM_053652.2 | (F) 5’-TAAGGTGTACACCACGCAGA-3’  (R) 5’-TGAGCTCTGAGAACTGTGCA-3’ | 319 bp |
| GATA-4 | NM_ 144730.1 | (F) 5’-GCTGCTGTGCCCATAGTG-3’  (R) 5’-AAAACGGAAGCCCAAGAA-3 | 184 bp |
| KDR | NM_013062.2 | (F) 5’-CGATGTCTCCTCCATCGTTT-3  (R) 5’-TTCCATCCGGAACAAATCTC-3 | 200 bp |
| NKX2.5 | NM_ 053651.2 | (F) 5’-CGGTGGAGCTGGACAAAGCC-3’  (R) 5’-TAGCGGCGGTTCTGGAACCA-3’ | 216 bp |
| Tbx2 | NM_001401806.1 | (F) 5’-CATCGCTGTCACTGCCTAC-3’  (R) 5’-CCGTCACGCTCCGGTTTA-3’ | 167 bp |
| Tbx5 | NM_001009964.1 | (F) 5’-GCAGGGAGGCAGATGTTT-3’  (R) 5’-GGCTCGGCTTTACCAGTT-3’ | 152 bp |
| VEGFA | NM_031836.3 | (F) 5’-CTCACCAAAGCCAGCACATA-3’  (R) 5’-AAATGCTTTCTCCGCTCTGA-3’ | 199 bp |
| VEGFC | NM_053653.2 | (F) 5’-TGTAAAACTTGCTGCTGCACATT-3’  (R) 5’-GAACGTCTAATAATTGAATGAACTTGTCT-3’ | 366 bp |
| vWF | NM_053889.1 | (F) 5′-GCGGTGTAAACGGACATCTC-3′  (R) 5′-ACAGGTTCGGGCATACTCAA-3′ | 232 bp |
| α-SMA | NM_031004.2 | (F) 5′-CGATAGAACACGGCATCATC-3′  (R) 5′-GGCAGGGACATTGAAGGTCT-3′ | 350 bp |
| β-actin | NM_031144.3 | (F) 5′-GTGTGACGTTGACAT-3′  (R) 5′-ACATCTGCTGGAAGGTG-3′ | 214 bp |

**
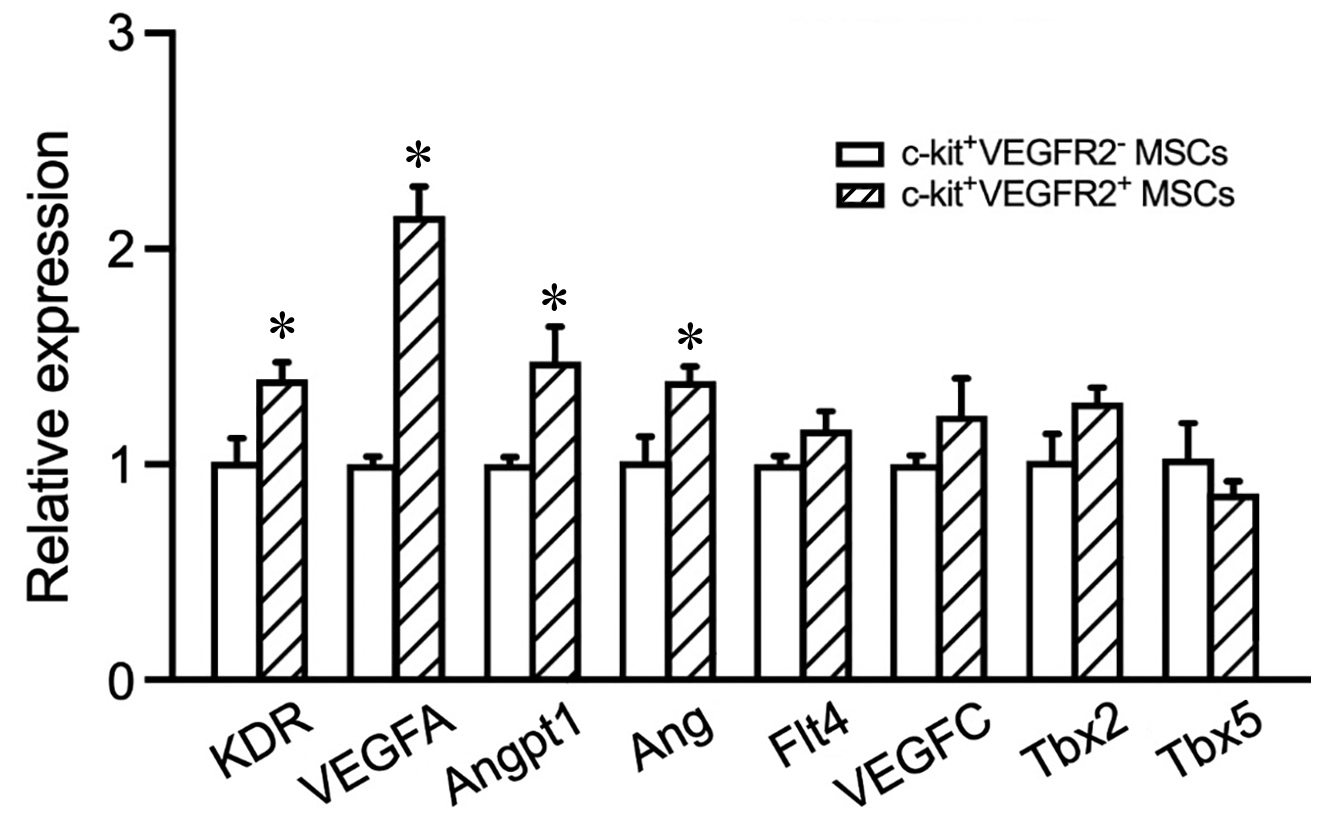
**

**Fig. S1** Expression of the genes related angiogenesis, lymphangiogenesis and myocardium development in c-kit^+^VEGFR-2^+^ MSCs. RT-PCR. **p* < 0.05 versus c-kit^+^VEGFR-2^-^ MSCs. n = 3


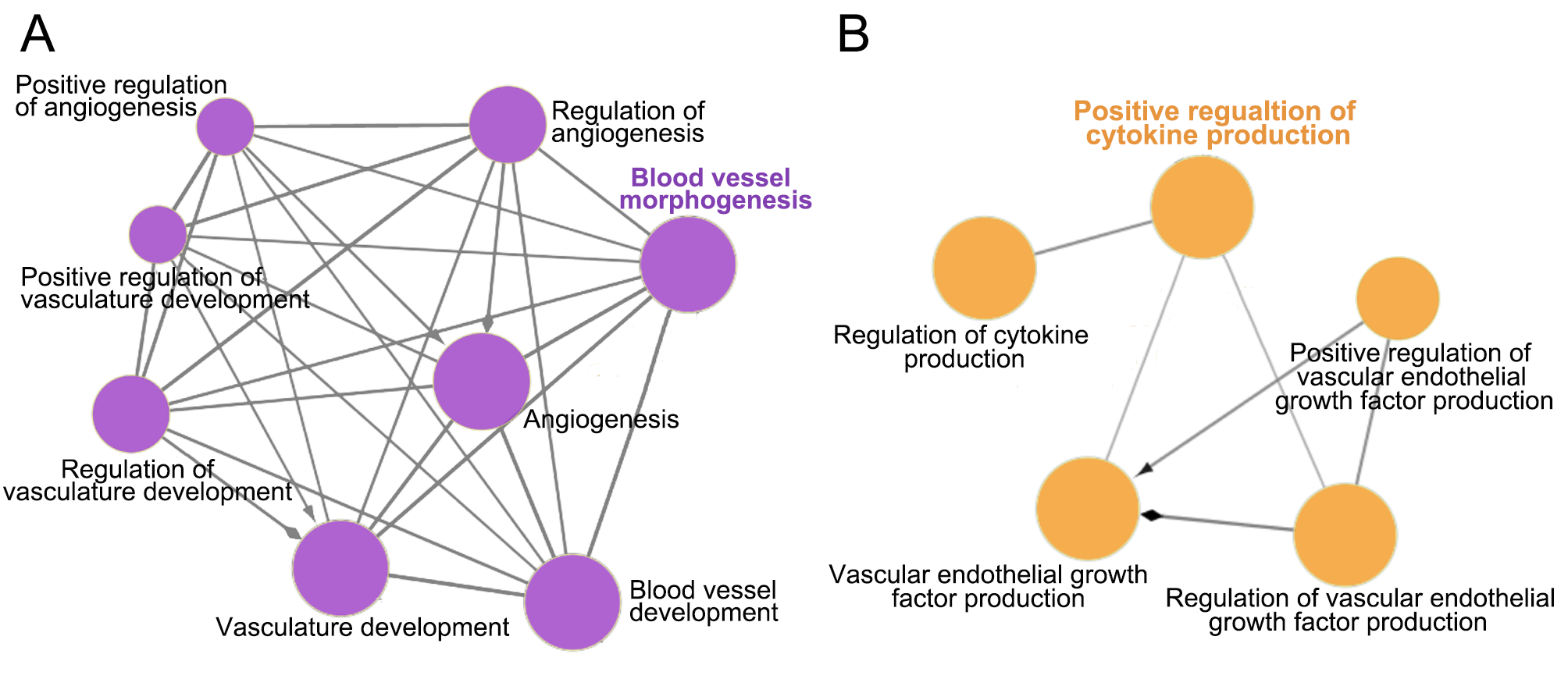


**Fig. S2** The first networks of regulating pathways of angiogenesis and cytokine production in c-kit^+^VEGFR-2^+^ MSCs. **A** The network of angiogenesis. **B** The network of cytokine production.

**
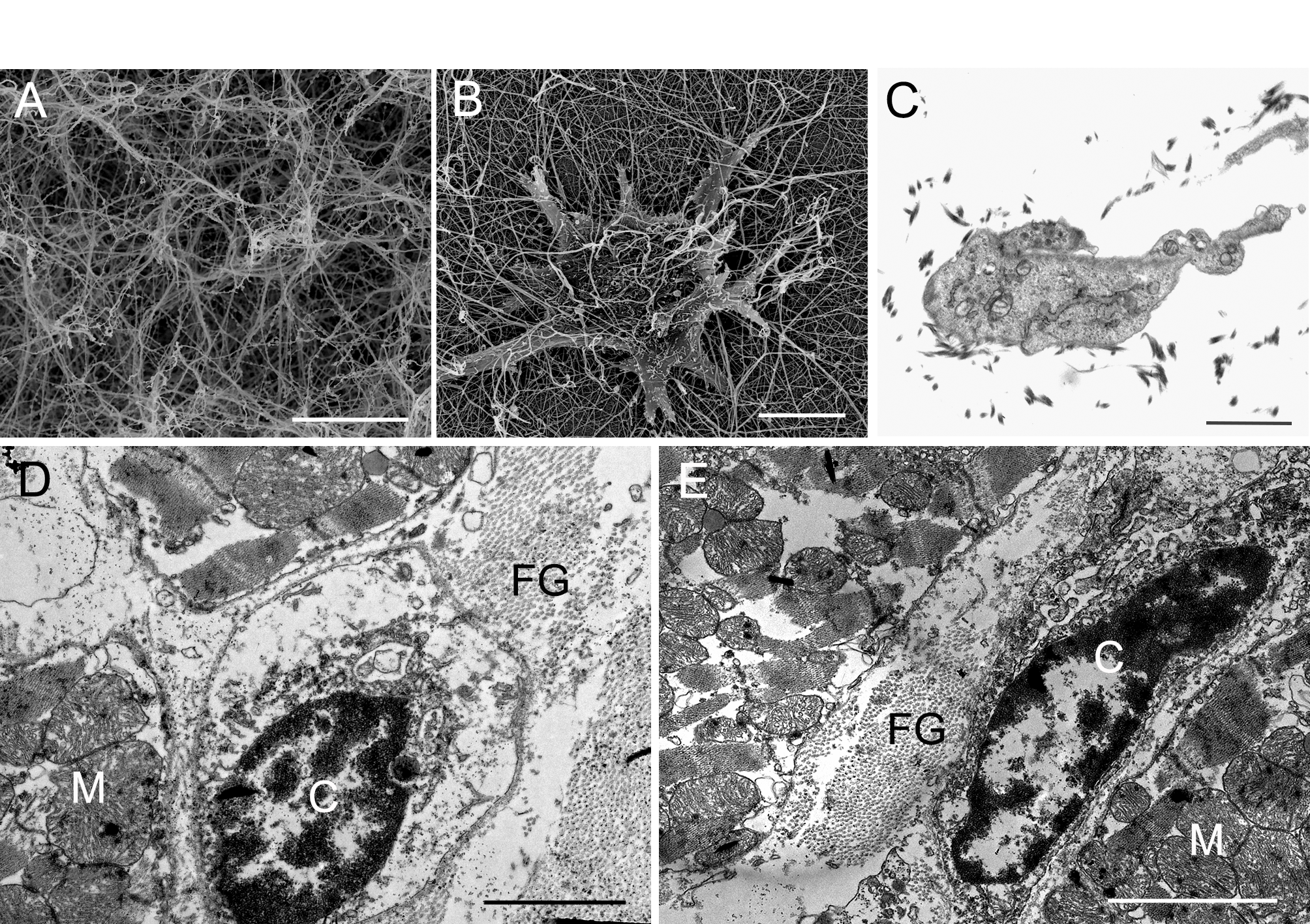
**

**Fig. S3** Electron micrographs of c-kit^+^VEGFR-2^+^ MSCs in fibrin gel. **A** The scanning electron micrograph of the fibrin nanofibres. **B** The scanning electron micrograph of the cell in the fibrin nanofibres. **C** The transmission electron micrograph of the cell in fibrin gel. **D**, **E** The transmission electron micrographs of the cells in fibrin gel at 2 h after transplantation into myocardium. C, c-kit^+^VEGFR-2^+^ MSC; FG, fibrin gel; M, myocardium. Scale bar = 10 μm (**A**), 2 μm (**B**–E).


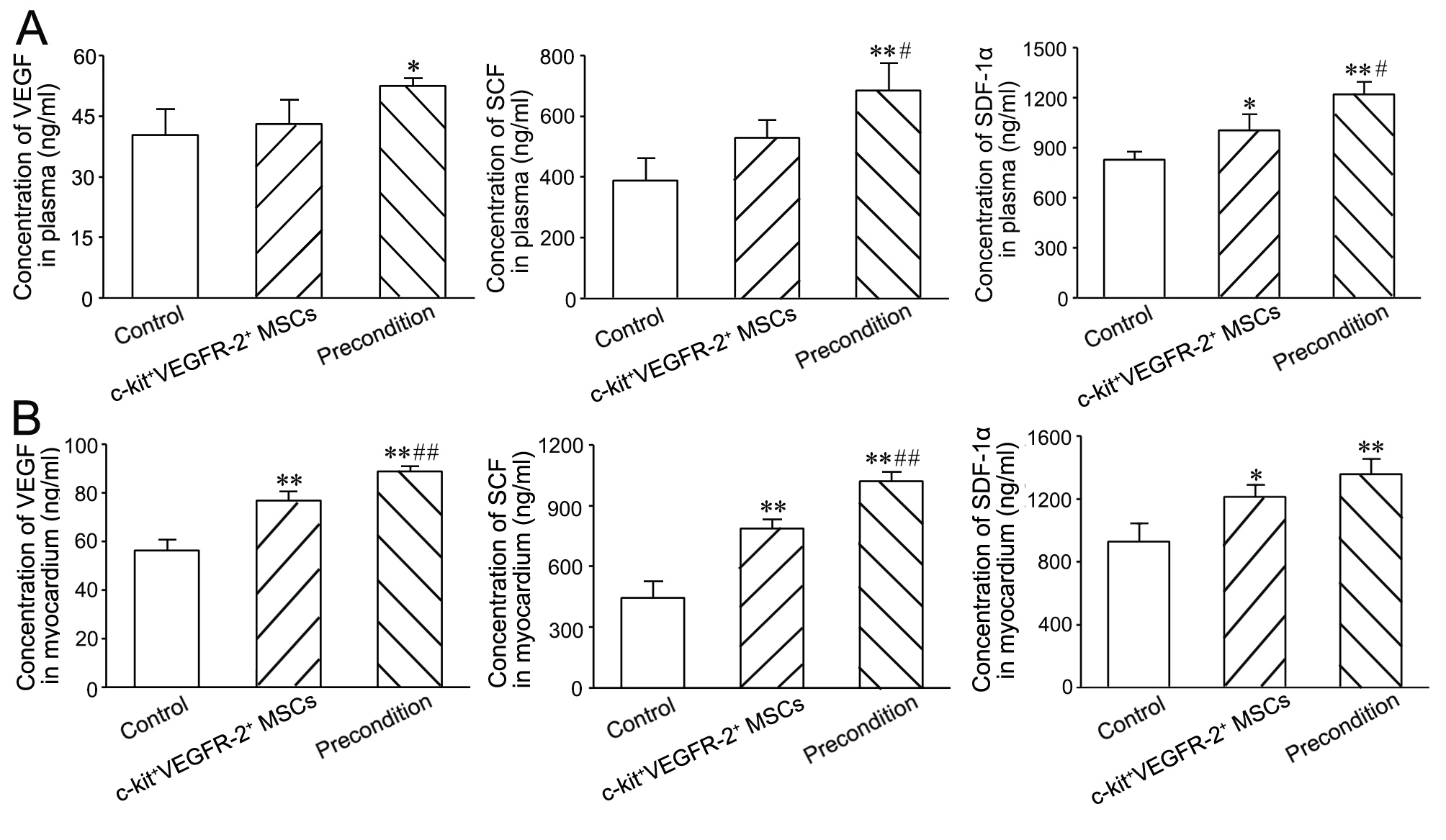


**Fig. S4** Concentration of paracrine factors at 1 week after transplantation. **A** Concentration of VEGF, SCF and SDF-1α in plasma. **B** Concentration of VEGF, SCF and SDF-1α in the peri-infarcted myocardium. **p* < 0.05, ***p* < 0.01 versus control group. #*p* < 0.05 and ##*p* < 0.01 versus c-kit^+^VEGFR-2^+^ MSC group. n = 6
